# Replication of biocompatible, nanotopographic surfaces

**DOI:** 10.1038/s41598-017-19008-z

**Published:** 2018-01-12

**Authors:** Xiaoyu Sun, Matt J. Hourwitz, Eleni M. Baker, B. U. Sebastian Schmidt, Wolfgang Losert, John T. Fourkas

**Affiliations:** 1 Department of Chemistry and Biochemistry, University of Maryland, College Park, MD 20742 USA; 2 Institute for Physical Science and Technology, University of Maryland, College Park, MD 20742 USA; 3 Department of Physics, University of Maryland, College Park, MD 20742 USA; 4 Center for Nanophysics and Advanced Materials, University of Maryland, College Park, MD 20742 USA; 5 Maryland NanoCenter, University of Maryland, College Park, MD 20742 USA

## Abstract

The ability of cells to sense and respond to nanotopography is being implicated as a key element in many physiological processes such as cell differentiation, immune response, and wound healing, as well as in pathologies such as cancer metastasis. To understand how nanotopography affects cellular behaviors, new techniques are required for the mass production of biocompatible, rigid nanotopographic surfaces. Here we introduce a method for the rapid and reproducible production of biocompatible, rigid, acrylic nanotopographic surfaces, and for the functionalization of the surfaces with adhesion-promoting molecules for cell experiments. The replica surfaces exhibit high optical transparency, which is advantageous for high-resolution, live-cell imaging. As a representative application, we demonstrate that epithelial cells form focal adhesions on surfaces composed of nanoscale ridges and grooves, and that the focal adhesions prefer to localize on the nanoridges. We further demonstrate that both F-actin and microtubules align along the nanoridges, but only F-actin aligns along the nanogrooves. The mass production of nanotopographic surfaces opens the door to the investigation of the effect of physical cues on the spatial distribution and the dynamics of intracellular proteins, and to the study of the mechanism of mechanosensing in processes such as cell migration, phagocytosis, division, and differentiation.

## Introduction

In physiological environments, cells are not only subjected to chemical signals, but also to physical interfaces that present various geometries and topographies. For instance, the phagocytosis of microparticles by macrophages depends on the shape and the orientation of the particles^1,2^. The activation of T cells can be initiated by contacting antigen-presenting dendritic cells, which are characterized by complex surface topographies such as filopodial protrusions, membrane ruffles, and invaginations^3^. The extracellular matrix (ECM), which is replete with collagen fibers that are hundreds of nanometers wide, has been demonstrated to be crucial in physiological processes, such as cell differentiation^4^, embryonic development^5^, immune response^6^, and wound healing^7^. In contrast, an aberrant ECM contributes to pathological conditions, such as rheumatoid arthritis^8^ and cancer metastasis^9,10^. Although certain proteins, such as actin and integrins, have been identified as playing a role in these processes, the effect of physical cues on the dynamics of these proteins and the mechanism of their mechanosensing remain elusive. Compared with commonly-used 2D chemical micropatterns on flat surfaces^11,12^, nanotopographies that resemble the physical features *in vivo* provide a more physiologically-relevant tool for studying cellular behaviors and intracellular dynamics, as well as for dissecting the mechanism of mechanosensing. For example, nanoridges^13^ and asymmetric microsawteeth^14^ have been demonstrated to guide cytoskeletal dynamics (esotaxis) and cell migration (microthigmotaxis). Micro- and submicro-pillars have been found to affect cellular rigidity sensing via biasing the localization of myosin filaments and focal adhesions^15^. Disordered nanodots^16^ have been shown to induce cell differentiation. With growing interest in the effect of physical cues, such as topography and curvature, on cell behavior and intracellular dynamics, methods for generating nanotopographic biomaterials are highly desirable.

A number of techniques have been developed for the generation of nanotopographic materials for biomedical applications. One such method is the replica molding of photolithographically-fabricated master surfaces using photocurable hydrogels^17^. However, some of the photoinitiators involved in the hydrogel photopolymerization have been found to be cytotoxic^18,19^, and the underlying topography can deform under the nanoNewton cellular traction force due to the compliance of the hydrogels^20^. Although the ECM and other biological microenviroments are compliant on the mesoscale, they are rigid at the nanoscale. It has been suggested that both convex- and concave-curvature-sensing proteins can stimulate actin polymerization^21^, so nanotopography that retains its original geometry is desirable for comparing how topography comparable to that of the ECM affects cytoskeletal dynamics. Injection molding has been used for the rapid creation of rigid, polymeric nanotopographic substrates^22^. However, the method is limited to molding low-aspect-ratio nanostructures, and the thick flash layer formed in the injection cavity presents a challenge for the use of the replicas in high-magnification cell imaging.

Here we report a method for the rapid generation of rigid, biocompatible nanotopographic materials that can be chemically functionalized with ease. By adding solvent to the polydimethylsiloxane (PDMS) prepolymer, we are able to mold complex nanotopographies with resolution as fine as ~25 nm. The resulting PDMS mold can also serve as a master for the replication of complementary nanotopographies. The acrylic resin used in the replica molding provides a platform for the subsequent surface functionalization for cell experiments. Resin formulations and processing conditions that prevent cytotoxicity in the replica surfaces are also examined. The molding resolution, versatility of surface functionalization, and potential applications of the nanotopographic surfaces are discussed.

## Materials and Methods

### Fabrication of master nanotopographic surfaces

To improve the adhesion of nanostructures, glass slides were treated with an oxygen plasma (Harrick plasma cleaner, PDC-32G) at 200 mTorr for 3 min, and immersed overnight in a solution composed of 4 mL of (3-acryloxypropyl)trimethoxysilane (SIA0200.0, Gelest), 10 mL of deionized water, and 196 mL of ethanol. The acrylate-functionalized slides were then rinsed in ethanol for 1 h and baked at 90 °C for 1 h. The acrylic resin was composed of 49 wt% tris (2-hydroxyethyl) isocyanurate triacrylate (SR368, Sartomer), 49 wt% dipentaerythritol pentaacrylate (SR399, Sartomer), and 2 wt% Lucirin TPO-L (Ciba). The resin was mixed overnight to ensure homogeneity. A drop of resin was applied to the acrylate-functionalized glass slide and the slide was mounted on a piezoelectric stage (Physik Instrumente). Master nanotopographic surfaces were fabricated via multiphoton absorption polymerization (MAP)^23^ using a commercial Ti:sapphire laser (Coherent Mira 900-F, 200 fs pulsed, 800 nm). The beam was focused on the sample through a 100× objective (NA 1.4). The power was 4 mW as measured at the sample. Upon completion of fabrication, the sample was developed in *N*,*N*-dimethylformamide and ethanol to remove the unpolymerized resin. Master nanograss^24^ was created by subjecting nanoridges to reactive ion etching (RIE) in a gas mixture of O_2_ (5 sccm) and CHF_3_ (15 sccm) at 150 W and 50 mTorr for 10 min.

### Solvent-assisted nanotransfer molding

The method of molding the nanotopographies is adapted from prior work^25^. Briefly, the master surface was functionalized with ethylenediamine (E26266, Sigma-Aldrich) and then reacted with perfluorooctadecanoic acid (L16837, Alfa Aesar) to reduce the surface energy and facilitate the release of cured PDMS. The hard PDMS mixture was composed of 1.7 g of vinyl PDMS prepolymer (VDT-731, Gelest), 9 μL of Pt catalyst (SIP6831.2, Gelest), 0.05 g of modulator (87927, Sigma-Aldrich), 0.5 g of hydrosilane (HMS-301, Gelest), and 1 g of hexane. The mixture was spin-coated on the master surface (1000 rpm, 40 s), allowed to sit at room temperature for 2 h, and prebaked at 60 °C for 1 h. Soft PDMS was prepared by mixing the base and curing agent (Sylgard 184, Dow Corning) in a 10:1 mass ratio. After degassing, the uncured soft PDMS prepolymer was poured onto the precured hard PDMS to form a layer of roughly 5 mm thick, and was then baked at 60 °C for 1 h. After curing, the composite PDMS was peeled off of the master surface. In the double-molding process, the first mold served as a master. The mold was treated with an oxygen plasma at 200 mTorr for 30 s. The mold was then transferred into a desiccator and was exposed to the vapor of (tridecafluoro-1,1,2,2,-tetrahydrooctyl)methyldichlorosilane (SIT8172.0, Gelest) for 1 h. The master was molded in the same manner as described above. Unless otherwise noted, the acrylic resin for replicating the nanotopographic surfaces was made by mixing 49 wt% tris (2-hydroxyethyl) isocyanurate triacrylate (SR368, Sartomer), 49 wt% ethoxylated trimethylolpropane triacrylate (SR499, Sartomer), and 2 wt% Lucirin TPO-L (Ciba). Replicas of nanotopographic surfaces were created by sandwiching a drop of acrylic resin between the mold and an acrylate-functionalized coverslip and then UV curing for 5 min (Blak-Ray, B-100AP, 100 W, 365 nm; samples were cured 254 mm from the source).

### Uniform coating of proteins

Replicas were soaked in ethanol and then in ultrapure water for 12 h each to allow the unreacted photoinitiator and byproducts of polymerization to diffuse out. For collagen IV coating, replicas were immersed in 25 μg/mL collagen (354233, Corning) in 0.05 M HCl atop ice for 1 h, and then rinsed two times by aspirating in and out ultrapure water. To image the coating using a confocal fluorescence microscope, the surfaces were treated with 0.5 mg/mL Alexa Fluor 594 NHS ester (A37572, Thermo Fisher Scientific) in 0.1 M NaHCO_3_ for 1 h, and then rinsed three times by aspirating in and out phosphate buffered saline (PBS). For fluorescent fibronectin coating, replicas were coated with 10 μg/mL HiLyte Fluor 488 fibronectin (FNR02-A, Cytoskeleton) in Hank’s balanced salt solution (HBSS) at 37 °C for 1 h and rinsed with three times by aspirating in and out HBSS.

### Surface characterization

Master and replica surfaces were imaged using a Hitachi S-4700 SEM. The surfaces were sputter-coated with Pt/Pd in argon plasma at 20 mA for 25 s (Cressington sputter coater 108). The height/depth of the structures was characterized by a Veeco Multimode AFM in tapping mode using aluminum-coated silicon probes (TAP300AL-G, Ted Pella).

A collagen-coated surface treated with Alexa Fluor 594 NHS ester was imaged in PBS using a Leica SP5 X confocal microscope with a 100× objective (NA 1.4) and a scanner zoom factor of 4. Images were obtained in a 512 × 512 pixel format. The fluorescent-fibronectin-coated surface was imaged in HBSS in the same manner. The orthogonal view is a single slice reconstructed from z-stack images.

### Cell culture, fixing, and staining

MCF10A cells were cultured in DMEM/F-12 medium (11330-057, Thermo Fisher Scientific) supplemented with 5% horse serum (26050-088, Invitrogen), 10 μg/mL insulin (12585-014, Thermo Fisher Scientific), 10 ng/mL EGF (AF-100-15, PeproTech), 0.5 μg/mL hydrocortisone (H0888, Sigma-Aldrich) and 100 ng/mL cholera toxin (C8052, Sigma-Aldrich). 2 mL of MCF10A cells with a density of 1 × 10^4^ cells/mL were plated on collagen-IV-coated 1.5-μm-spaced ridges and were incubated at 37 °C overnight. To stain tubulin and actin, the cells were fixed with 4% paraformaldehyde in DMEM/F12 containing 50 mM PIPES for 10 min and permeabilized with 1% Saponin for 10 min. The samples were blocked in 1% bovine serum albumin (BSA) for 1 h, incubated with 1:400 anti-tubulin primary antibody (MAB1864, rat host, EMD Millipore) at 4 °C overnight, and washed three times in PBS for 5 min each. Next, the samples were incubated at room temperature for 1 h with 1:250 goat anti-rat IgG secondary antibody conjugated to Alexa Fluor 568 (A-11077, Thermo Fisher Scientific), and 1:200 phalloidin conjugated to Alexa Fluor 488 (A12379, Thermo Fisher Scientific), and then incubated at room temperature for another 15 min with 1:40000 DAPI (D1306, Thermo Fisher Scientific). Finally, the samples were washed three times in PBS for 5 min each. A standard protocol was adopted to stain paxillin and actin. Briefly, the cells were fixed with 4% paraformaldehyde in PBS and permeablized with 0.2% Triton X-100 in PBS. The samples were blocked in 1% BSA for 1 h, incubated at 4 °C overnight with 1:500 phospho-paxillin pTyr118 primary antibody (44–722 G, rabbit host, Thermo Fisher Scientific), and then incubated at room temperature for 1 h with 1:250 goat anti-rabbit IgG secondary antibody conjugated to Alexa Fluor 568 (A-11011, Thermo Fisher Scientific) and 1:200 phalloidin conjugated to Alexa Fluor 488 (A12379, Thermo Fisher Scientific). Finally, the samples were imaged using a Perkin Elmer spinning-disk confocal microscope with a 100× objective (NA 1.49).

### Evaluation of cytotoxicity

Resins were made by mixing SR368, SR499, and photoinitiators at the weight percentages listed in Table 1. The systematic names of the listed photoinitiators are (going from the top to the bottom of Table 1): 1-hydroxycyclohexyl-phenyl-ketone, 2-benzyl-2-(dimethylamino)-4′-morpholinobutyrophenone, 2,2-dimethoxy-2-Phenylacetophenone, phenylbis(2,4,6-trimethylbenzoyl)phosphineoxide, 2,4,6-trimethylbenzoylphenyl phosphinate. 6.4-μm-spaced ridges were replicated on acrylate-functionalized coverslips and coated with collagen IV. Collagen-coated glass and tissue-culture-treated culture dish (CLS430165, Corning) served as control surfaces. MCF10A cells were plated in four separated 18 uL drops on each surface at a density of 1.67 × 10^6^ cells/mL and were given 1 h to adhere to the surfaces in the incubator. Non-adherent cells were then washed off and fresh medium was added to the plate. The cells were cultured on the surfaces for 48 h before being washed with PBS and incubated in 0.25% trypsin (25200056, Invitrogen) at 37 °C for 10 min. The culture medium, washing buffer, and cell-enriched trypsin were collected and centrifuged at 200 g for 4 min. Cells were then resuspended in 50 uL of PBS and incubated with 50 uL of 0.4% Trypan Blue (15250–061, Thermo Fisher Scientific) at room temperature for 5 min. Finally, cells were loaded into a hemocytometer. The numbers of dead (blue) and live cells were counted under a microscope.

**Table 1:** MCF10A cell viability averaged over three experiments on nanoridge surfaces replicated with various photoinitiators.

Photoinitiator/Control	Concentration (wt%)	Viability (%)
Irgacure 184	1.12	95
Irgacure 369	0.92	98
Irgacure 651	1.19	96
Irgacure 819	0.59	97
Irgacure TPO-L	2.01	96
Benzoin methyl ether	1.32	68
Collagen-coated glass	NA	96
Tissue culture plate	NA	96

### Image analysis

A custom MATLAB (MathWorks) script was used to determine the angle between the ridges and the horizontal axis from maximum intensity projection of the z-stack images. The 3D stack was then rotated by this angle using ImageJ^26^ plugin VolumeViewer to create an average image of the cross section along the ridges using the fluorescence intensity as weight for the voxels.

### Data Availability

All data generated or analyzed during this study are included in this article and its Supplementary Information files.

## Results

**Figure 1:**
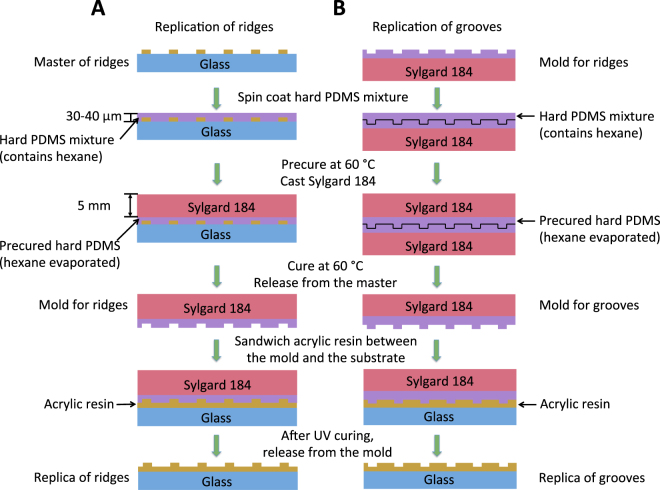
Schematic of replication processes for nanoridges (**A**) and nanogrooves (**B**).

In our method, a master surface is fabricated using MAP^27^. The master surface is then functionalized with perfluorocarbon to reduce the surface energy and to facilitate the subsequent release of the PDMS mold. In the solvent-assisted nanotransfer molding, hexane-containing hard PDMS prepolymer^28^ is cast on the perfluorocarbon-passivated master surface containing the desired nanotopography. Hexane serves as a solvent to reduce the viscosity and the surface tension of the hard PDMS prepolymer, and thus to assist the prepolymer’s penetration into gaps and voids. After precuring at 60 °C, a thick layer of soft PDMS prepolymer is poured on top of the hard PDMS and cured again at 60 °C. Next, a drop of acrylic resin is sandwiched between the PDMS mold and the acrylate-functionalized cover glass, and then is photocured for 5 min. The resulting replica has nanotopography that is identical to that on the master surface (Fig. 1A). The complementary (negative) nanotopography can be produced by using the negative PDMS mold as a master for a new round of molding (Fig. 1B).

**Figure 2:**
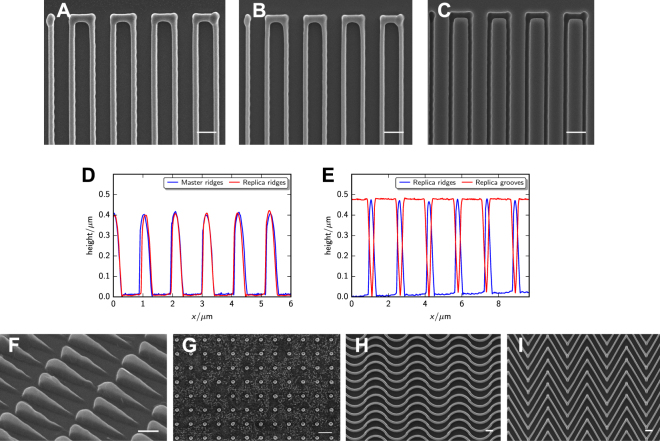
Characterization of the fidelity of the solvent-assisted nanotransfer molding. (**A–C**) SEM images of master ridges (**A**), replica ridges (**B**), and replica grooves (**C**). The negative topography **C** in the juxtaposition has been flipped horizontally for consistency. (**D**,**E**) Sectional AFMs of 1-μm-spaced master (blue) and replica (red) ridges (**D**), and 1.5-μm-spaced replica ridges (blue) and grooves (red) (**E**). (**F–I**) SEM images of asymmetric sawteeth (**F**), nanoposts (**G**), curved ridges (**H**), and kinked ridges (**I**). Scale bars, 1.5 μm (**A–C**), 1 μm (**F**), and 2 μm (**G–I**).

We examined the fidelity of the molding process by comparing the nanotopography of master and replica substrates using scanning electron microscopy (SEM) and atomic force microscopy (AFM). The widths of the master ridges (Fig. 2A), replica ridges (Fig. 2B), and replica grooves (Fig. 2C) are all ~350 nm, with no discernible difference between master and replica. AFM profiles show that the ridge heights and groove depths are also replicated accurately (Fig. 2D, Supplementary Fig. 1A,B, 400-nm-tall master and replica ridges; Fig. 2E, Supplementary Fig. 1C,D, 470-nm-tall replica ridges and 470-nm-deep replica grooves). Using this method, we are able to replicate a diverse range of topographies, representative examples of which are shown in Fig. 2F-I. Asymmetric microsawteeth (Fig. 2F) have been demonstrated to induce unidirectional cell migration through esotaxis^14^. Nanoposts with a diameter of ~500 nm (Fig. 2G) are on the same size scale as many bacteria, rendering these structures a useful tool for studying cytoskeletal dynamics involved in frustrated endocytosis and invagination. Curved (Fig. 2H) and kinked (Fig. 2I) nanoridges mimic collagen fibers and nodes in the branched collagen network *in vivo*, respectively, providing a platform for the systematic study of how curvature and branching angles affect intracellular dynamics.

**Figure 3:**
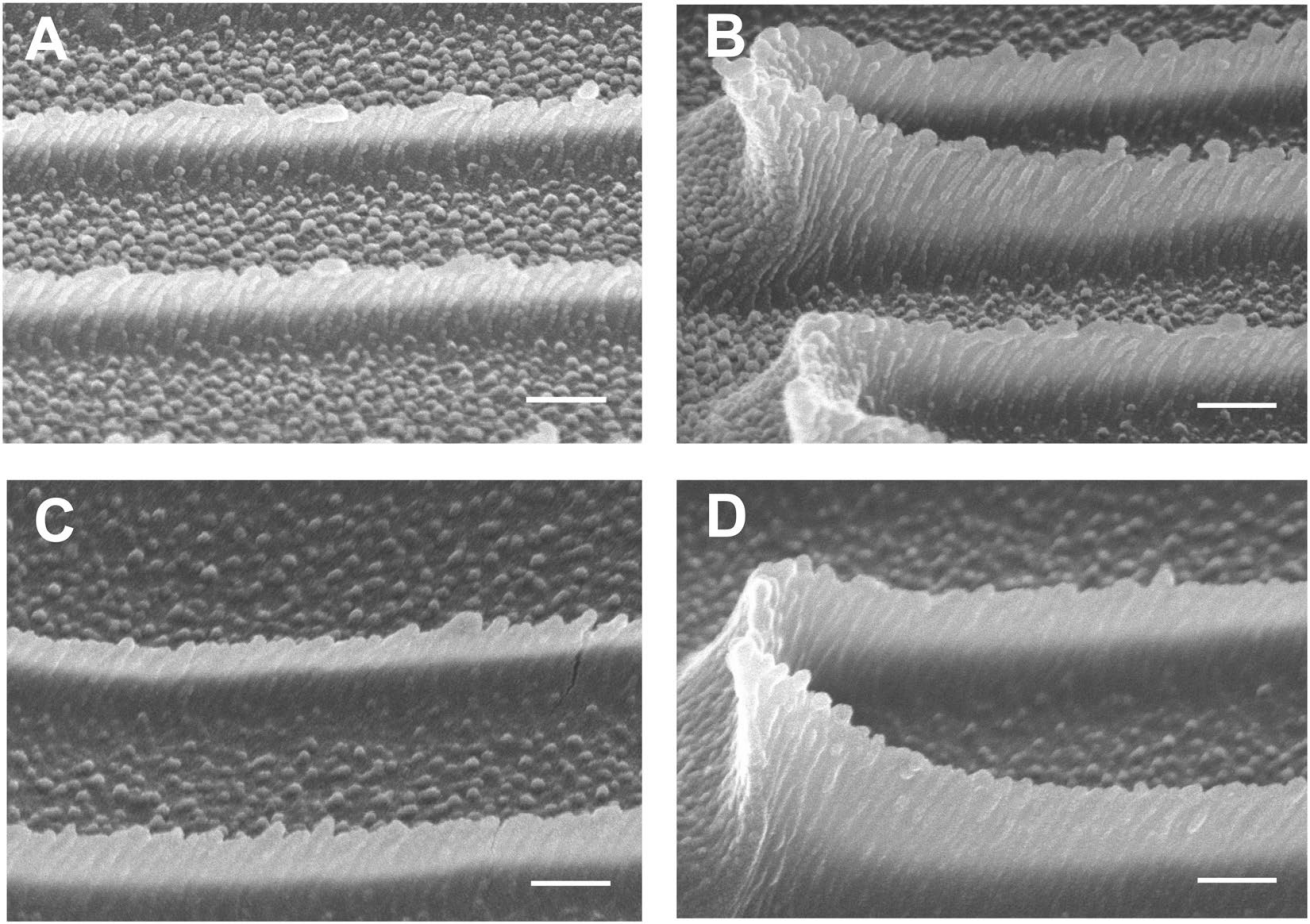
Replication of nanograss. SEM images of the master (**A**,**B**) and replica (**C**,**D**) nanograss. Scale bars, 200 nm.

To assess the resolution of the solvent-assisted nanotransfer molding, it would be desirable to mold nanostructures that are smaller and more closely-packed than the aforementioned nanotopographies. However, the typical width of a MAP-fabricated voxel is ~300 nm. To test whether our method is capable of molding features smaller than 300 nm, an alternative master nanotopography was needed. Therefore, nanograss with a thickness and spacing of ~25 nm (Fig. 3A,B) was used to investigate the resolution. This nanograss was created by subjecting the nanoridges to RIE. As shown in Fig. 3, the replica nanograss (Fig. 3C,D) exhibits thickness and spacing that are similar to those of the master nanograss (Fig. 3A,B), suggesting that the resolution of the molding is at least as fine as 25 nm.

**Figure 4:**
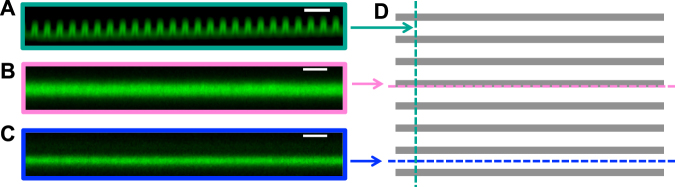
Functionalization of nanoridges with collagen IV. (**A–C**) Side-view images of the fluorescent-collagen-coated nanoridges reconstructed from z-stack confocal images. The planes are perpendicular to the surface in the position of the dashed lines in (**D**) of the color of the image borders. (**D**) Top-view schematic of nanoridges with grey representing the ridges. Scale bars, 3 μm.

We functionalized the nanoridges with collagen IV to render the surfaces adherent to mammalian cells such as MCF10A human breast epithelial cells and HL60 neutrophil-like cells. The exposed amine groups on collagen react naturally with surface acrylate groups through Michael addition^29^. To characterize the collagen coating, we treated the collagen-IV-coated nanoridges with NHS Alexa 594, which fluorescently labels the protein but not the acrylic polymer (Fig. 4). Figure 4A shows the average cross section reconstructed from confocal z-stack images, which starts below the bottom surface and ends above the tops of the nanoridges. The uniform fluorescence delineates the contour of the nanoridges, whereas the bulk polymer region remains dark. This phenomenon indicates that the nanoridges are coated conformally with collagen. The fluorescence along the sides of the ridges and the middles of the trenches further confirms the conformal nature of the collagen coating (Fig. 4B,C). [Note that the thickness of the coating is exaggerated in the axial direction due to the elongated point-spread function of the microscope.] Similar results were obtained on nanoridges coated with fluorescently labeled fibronectin (Supplementary Fig. 2), indicating the functionalization of nanotopographies replicated in acrylic resin is not protein-specific. Any protein/peptide with surface amine and/or thiol groups is expected to exhibit good adhesion. The curing time of the replica can also be tuned to adjust the density of residual acrylate groups on the polymer surface available for the subsequent protein coating^29^.

**Figure 5:**
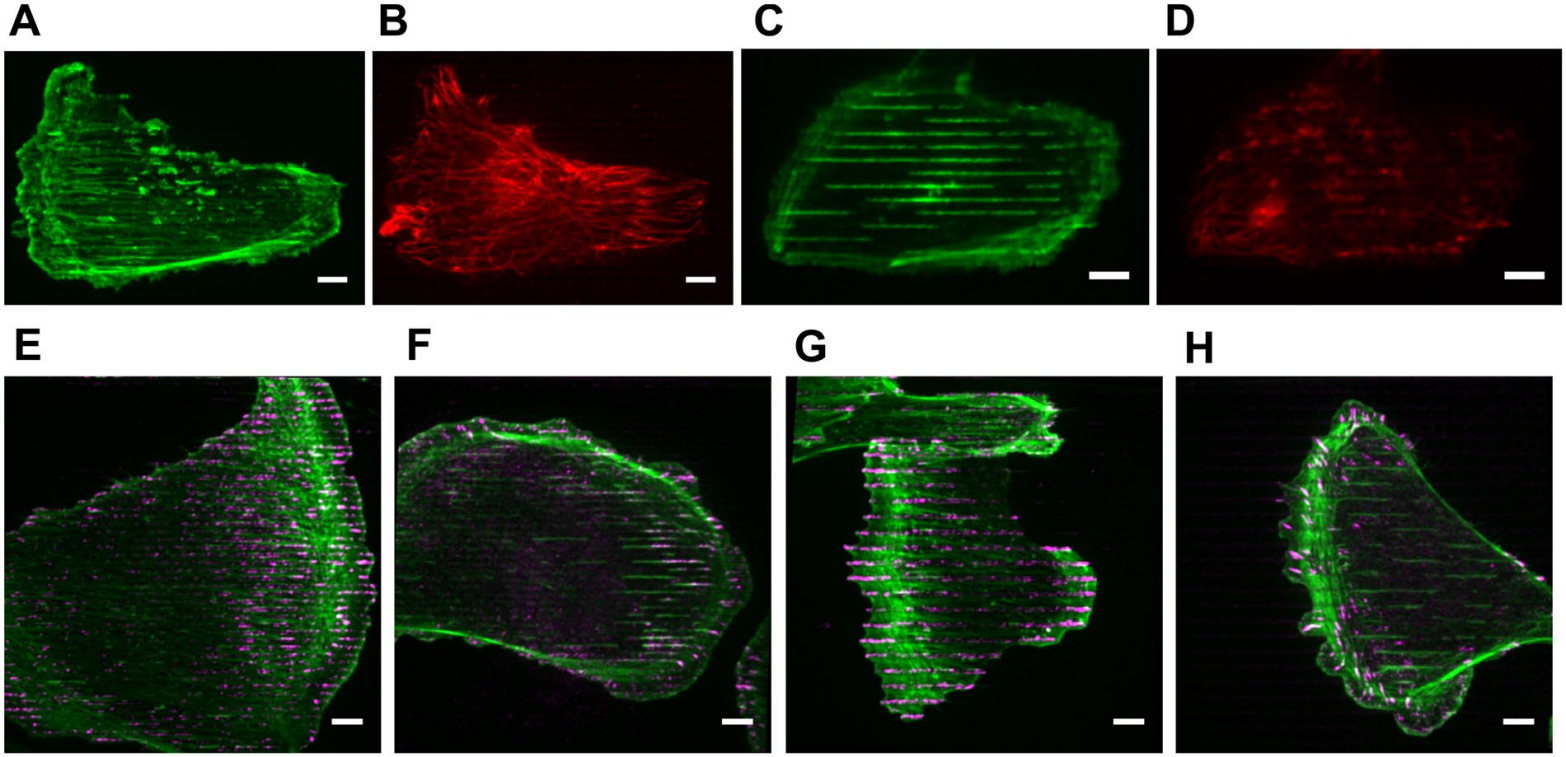
Cytoskeletal alignment and focal adhesion distribution on nanoridges and nanogrooves. (**A–H**) Maximum intensity projection of confocal z-stacks that go through the entire ridge/groove volume. The nanoridges/grooves are horizontally oriented. (**A–D**) Actin (**A**,**C**) and tubulin (**B**,**D**) stain of one cell on 2-μm-spaced nanoridges (**A**,**B**) and another cell on 2-μm-spaced nanogrooves (**C**,**D**). (**E–H**) Actin (green) and paxillin (magenta) stain of cells on nanoridges (**E**, 1.5-μm-spaced; (**G**) 3-μm-spaced) and nanogrooves (**F**, 1.5-μm-spaced; (**H**) 3-μm-spaced). Scale bars, 6 μm.

To investigate how replicated nanotopography affects cytoskeletal alignment and the distribution of focal adhesions, we plated individual MCF10A cells on collagen-IV-coated nanoridges and nanogrooves, and fixed the cells after 12 hours. Cells tend to elongate along the 1.5-μm- and 2-μm-spaced ridges and grooves (Fig. 5A–F). Both F-actin (Fig. 5A) and microtubules (MTs) (Fig. 5B) form long fibers that align along the ridges. We observed strong alignment of MTs but not of F-actin near the trailing edge of the same cell (cf. the right part of the cell in Fig. 5A,B), indicating that MTs align naturally along the ridges, rather than being dragged into alignment by F-actin. Inside the grooves, however, F-actin primarily forms long streaks (Fig. 5C), whereas MTs barely appear (Fig. 5D). Differences in the localization of focal adhesions (FAs) are also observed between cells on the ridges and cells on the grooves, although FAs form on both types of nanotopography (Fig. 5E–H). FAs prefer to localize on most of the ridge area covered by the cell, especially near the cell periphery (Fig. 5E,G). However, FAs either only localize in limited numbers inside the grooves that are near the cell periphery (Fig. 5F), or barely appear inside the grooves (Fig. 5H). Even though F-actin forms streaks in the grooves covered by the interior cellular region, FAs still avoid those groove areas (Fig. 5H).

The use of photoinitiators, which are incorporated into the polymer upon reaction and are then no longer photoactive, is advantageous over the use of photosensitizers such as Rose Bengal^30,31^, which retain their ability to generate radicals after polymerization. To ensure the creation of non-cytotoxic replicas of nanotopographies, we molded 6.4-μm-spaced nanoridges using acrylic resins prepared with a range of different photoinitiators. To remove potentially toxic unreacted photoinitiator molecules and byproducts of polymerization, the surfaces were soaked in ethanol and water before being coated with collagen IV and plated with MCF10A cells. Table 1 lists the photoinitiators that were tested, with the corresponding minimum concentrations required for the faithful replication of nanoridges, along with the average viability of MCF10A cells 48 hours after being plated. Collagen-IV-coated glass and tissue-culture-treated culture dishes served as controls. Also included as a reference is a collagen-IV-coated, nanoridge substrate replicated with 2 wt% TPO-L, the same photoinitiator/concentration used to replicate the topographies shown in Figs 1 and 2. We found that cell viabilities were above 95% on almost all the surfaces, except for ones replicated with benzoin methyl ether as the photoinitiator (Table 1, Supplementary Fig. 3). On some of the surfaces, the cell viabilities were even higher than those on the control surfaces. It is worth noting, however, that these data were not obtained under imaging conditions. Free radicals could be created during imaging by any residual photoinitiators, which may render the surface toxic to the cells. We have never observed this phenomenon, but in the event of any cytotoxicity during imaging the replication process should be optimized for the specific imaging conditions required for any given application.

## Discussion

We have demonstrated a method for the mass production of biocompatible, nanotopographic surfaces. The method is also capable of replicating complementary nanotopographies. The acrylic resin employed in the replica molding not only facilitates the subsequent surface functionalization for biological and biomedical applications, but also results in a rigid and highly transparent nanotopographic coating that can be used for high-resolution, live-cell imaging. We have previously found that the Young’s modulus of this acrylic polymer that was cured with UV light is 2 GPa^32^. Cells typically deform substrates that have a stiffness in the range of tens of kiloPascals or less^33^, so our replica surfaces are rigid enough to resist the cellular traction forces and to maintain the original topographies. 2 GPa is comparable to the rigidity of collagen fibers^34^, making the nanotopographic surfaces relevant to *in vivo* processes. The method is also applicable to replicating nanotopography that covers a large area, allowing specific proteins involved in mechanosensing and mechanotransduction pathways to be analyzed by techniques such as SDS-PAGE and enzyme-linked immunosorbent assays. In the work reported here the master is created using either MAP or RIE, but our replication technique is compatible with a wide variety of methods for the production of masters. Each master can be molded numerous times, and the resulting PDMS mold can be used more than 30 times without discernible damage. Each replica can also serve as a master and be molded repeatedly through the same process, allowing the production of a virtually unlimited number of replicas. In addition to acrylic resins, a broad range of other materials can be used for replication, although other materials require different surface-functionalization chemistries.

The theoretical resolution limit of nanotransfer molding is determined by the average chemical bond length and distance between crosslinks in hard PDMS^35^, as well as by the interaction between the master and the hard PDMS surfaces^36^. The increased wettability of hard PDMS prepolymer on the master surface and the increased crosslink density of the cured hard PDMS result in an improved molding resolution. The formulation and curing temperature of the hard PDMS prepolymer could be further tuned towards an even higher crosslink density. To increase the wettability of the hard PDMS, the molecular composition and the roughness of the hard PDMS and master surfaces need to be considered. In all the molding experiments we performed, the master surfaces were coated with fluorocarbon to reduce the surface energy, facilitating the release of the PDMS mold. However, a low-energy surface is associated with a low wettability, which prevents the hard PDMS prepolymer from conforming perfectly to the nanotopography. On a low-energy surface, the cosine of the contact angle increases linearly as the surface tension of the liquid decreases^37^. Therefore, adding a solvent to the hard PDMS prepolymer may reduce the surface tension and improve the conformability. Hexane possesses low surface tension (γ = 18.4 mN/m at 20 °C) and low viscosity (0.67 cp at 25 °C). The fact that hexane is miscible with PDMS prepolymer^38^ prevents phase separation of the mixture, thus makes this solvent an ideal candidate for improving the molding resolution. Limited by the available smallest features on the master nanotopography, we demonstrated a molding resolution of ~25 nm with nanograss (Fig. 3). However, the actual resolution could be even finer. When the features to be molded approach the molecular scale, the thickness of the anti-adhesion coating needs to be considered, as this coating will influence the geometry of the underlying nanotopography. Fluorocarbon molecules with a shorter chain length can be used, provided that the resultant surface energy is low enough to release the PDMS mold. Additionally, in our attempts to mold nanotopographies with high-curvature features, such as concentric rings and sinusoidal waves, the PDMS mold tends to stick to the high-curvature features. Separating the mold from the master while the two are immersed in a solvent that does not swell PDMS (such as methanol) substantially alleviates the sticking phenomenon, presumably due to a reduction in surface adhesion^39^ and roughness^40^.

An important advantage of using acrylic resin as the replica-molding material lies in the unreacted acrylate groups on the polymer surface, which can react readily with the amine and/or thiol groups exposed on a protein/peptide, allowing the nanotopographies to be functionalized with various biomolecules for cell experiments (Fig. 4, Supplementary Fig. 2). Because most mammalian cells require a substrate that is chemically similar to the ECM to form integrin-mediated FAs, substrates that are compatible with surface functionalization would be beneficial for studies of the effects of physical cues on mammalian cells. For instance, we have functionalized nanoridges with fibronectin and B cell receptor ligand to study the contact guidance of human tumor-associated fibroblasts (TAFs)^41^ and the activation of B cells^42^, respectively. We found that the TAFs and their actin stress fibers align along the nanoridges, and that the actin-wave dynamics and calcium signaling in B cells are modulated by the ridge spacing. In the present study, we demonstrate that MCF10A epithelial cells adhere to and align along the nanoridges/grooves (Fig. 5A–F). The paxillin stain indicates that the FAs form microdomains preferentially on the nanoridges (Fig. 5E,G). The fact that paxillin is a downstream component of the integrin-mediated signaling pathways suggests that the same localization may be observed with integrins. Because integrin activation is related to its unfolding, it would be interesting to investigate how nanotopography affects the conformational change of integrin, as well as the downstream pathways such as calcium signaling. Rather than coating the surface with collagen/fibronectin to enhance cell-substrate adhesion, coating the nanotopographic surface with BSA or concanavalin A can decouple the influence of physical cues from that of substrate-adherent ligand on the activation of integrin. Furthermore, the unreacted acrylate groups on the polymer surface also allow the nanotopography to be functionalized with other molecules, such as perfluorocarbons (section 2.2) and polyethylene glycol, to study non-integrin-mediated cell adhesion or migration. For instance, the collective migration of *Dictyosteliuim discoideum* cells has been found to be substantially affected by surface adhesivity^43^. Because the nanotopography fosters reproducibly controlled cytoskeletal dynamics (esotaxis)^13,14^, coupling nanotopography with different surface adhesivity may reveal how the actomyosin cortex coordinates with the weak adhesion molecules to regulate the cell-substrate and cell-cell adhesion.

The replicated nanotopographic surfaces may find use in an even broader range of fields. For instance, as shown in Fig. 5B, MTs naturally align along the nanoridges. Therefore, nanotopographic surfaces can be used to study activities related to MT alignment, such as cell division and intracellular trafficking. Additionally, given that our method possesses a molding resolution of ~25 nm or even higher, surfaces coated with nanoparticles and nanowires can be replica-molded with acrylic resin, providing nanotopography with features that approach molecular scale. These small features can be exploited to study the dynamics of curvature-sensing proteins such as the BAR-domain proteins. Although we only demonstrate single-cell studies on the nanotopographic surfaces, these surfaces can also be applied to study the effects of physical cues on cell sheets, tissues or even organoids, to elucidate the mechanisms of mechanosensing in processes such as embryonic development, immune responses, wound healing, and cancer metastasis.

## Conclusions

In conclusion, we have demonstrated a method for the replication of biocompatible, complementary nanotopographies and for the functionalization of the topographies with adhesion-promoting molecules. This approach may be an attractive alternative to other methods in terms of simplicity, low cost, the range of materials that can be used, and the ease of surface functionalization. As such, this approach should facilitate the investigation of phenomena such as the effects of physical cues on the dynamics of intracellular proteins and the role of mechanosensing in processes such as cell migration, phagocytosis, division, and differentiation. These nanotopographic surfaces can be applied to studies on single cells, as well as cell sheets, tissues and organoids, to help elucidate mechanisms in pathology and provide diagnostics for diseases.

## Electronic supplementary material

Supplementary information

## References

[1] Champion JA, Mitragotri S (2006). Role of target geometry in phagocytosis. Proc. Natl. Acad. Sci. USA.

[2] Champion JA, Mitragotri S (2009). Shape Induced Inhibition of Phagocytosis of Polymer Particles. Pharm. Res..

[3] Mempel TR, Henrickson SE, von Andrian UH (2004). T-cell priming by dendriticcells in lymph nodes occurs in three distinct phases. Nature.

[4] Watt FM, Huck WTS (2013). Role of the extracellular matrix in regulating stem cell fate. Nat. Rev. Mol. Cell Biol..

[5] Rozario T, DeSimone DW (2010). The Extracellular Matrix In Development and Morphogenesis: A Dynamic View. Dev. Biol..

[6] Lämmermann T (2013). Neutrophil swarms require LTB4 and integrins at sites of cell death *in vivo*. Nature.

[7] Maquart FX, Monboisse JC (2014). Extracellular matrix and wound healing. Pathol. Biol..

[8] Sorokin L (2010). The impact of the extracellular matrix on inflammation. Nat. Rev. Immunol..

[9] Provenzano PP (2006). Collagen reorganization at the tumor-stromal interface facilitates local invasion. BMC Med..

[10] Oudin MJ (2016). Tumor Cell–Driven Extracellular Matrix Remodeling Drives Haptotaxis during Metastatic Progression. Cancer Discov..

[11] Kilian KA, Bugarija B, Lahn BT, Mrksich M (2010). Geometric cues for directing the differentiation of mesenchymal stem cells. Proc. Natl. Acad. Sci..

[12] Elliott H (2015). Myosin II controls cellular branching morphogenesis and migration in three dimensions by minimizing cell-surface curvature. Nat. Cell Biol..

[13] Driscoll MK, Sun X, Guven C, Fourkas JT, Losert W (2014). Cellular Contact Guidance through Dynamic Sensing of Nanotopography. ACS Nano.

[14] Sun X (2015). Asymmetric nanotopography biases cytoskeletal dynamics and promotes unidirectional cell guidance. Proc. Natl. Acad. Sci..

[15] Ghassemi S (2012). Cells test substrate rigidity by local contractions on submicrometer pillars. Proc. Natl. Acad. Sci..

[16] Dalby MJ (2007). The control of human mesenchymal cell differentiation using nanoscale symmetry and disorder. Nat. Mater..

[17] Kim D-H (2010). Nanoscale cues regulate the structure and function of macroscopic cardiac tissue constructs. Proc. Natl. Acad. Sci..

[18] Williams CG, Malik AN, Kim TK, Manson PN, Elisseeff JH (2005). Variable cytocompatibility of six cell lines with photoinitiators used for polymerizing hydrogels and cell encapsulation. Biomaterials.

[19] Sabnis A, Rahimi M, Chapman C, Nguyen KT (2009). Cytocompatibility studies of an *in situ* photopolymerized thermoresponsive hydrogel nanoparticle system using human aortic smooth muscle cells. J. Biomed. Mater. Res. A.

[20] Kim D-H (2006). Guided Three-Dimensional Growth of Functional Cardiomyocytes on Polyethylene Glycol Nanostructures. Langmuir.

[21] Peleg B, Disanza A, Scita G, Gov N (2011). Propagating Cell-Membrane Waves Driven by Curved Activators of Actin Polymerization. PLoS ONE.

[22] Gadegaard N, Mosler S, Larsen NB (2003). Biomimetic Polymer Nanostructures by Injection Molding. Macromol. Mater. Eng..

[23] LaFratta CN, Fourkas JT, Baldacchini T, Farrer RA (2007). Multiphoton Fabrication. Angew. Chem. Int. Ed..

[24] Qin, S. The applications of multiphoton absorption polymerization (2013).

[25] Kang H, Lee J, Park J, Lee HH (2006). An improved method of preparing composite poly(dimethylsiloxane) moulds. Nanotechnology.

[26] Schneider CA, Rasband WS, Eliceiri KW (2012). NIH Image to ImageJ: 25 years of image analysis. Nat. Methods.

[27] Fourkas, J. T. Fundamentals of Two-Photon Fabrication. In *Three-Dimensional Microfabrication Using Two-photon Polymerization* (ed. Baldacchini, T.) 45–61 (William Andrew Publishing, 2016).

[28] Schmid H, Michel B (2000). Siloxane Polymers for High-Resolution, High-Accuracy Soft Lithography. Macromolecules.

[29] Li L (2008). Binary and Gray-Scale Patterning of Chemical Functionality on Polymer Films. J. Am. Chem. Soc..

[30] Pitts JD (2002). New Photoactivators for Multiphoton Excited Three-dimensional Submicron Cross-linking of Proteins: Bovine Serum Albumin and Type 1 Collagen. Photochem. Photobiol..

[31] Basu S, Rodionov V, Terasaki M, Campagnola PJ (2005). Multiphoton-excited microfabrication in live cells via Rose Bengal cross-linking of cytoplasmic proteins. Opt. Lett..

[32] Bayindir Z (2005). Polymer microcantilevers fabricated via multiphoton absorption polymerization. Appl. Phys. Lett..

[33] Yip AK (2013). Cellular Response to Substrate Rigidity Is Governed by Either Stress or Strain. Biophys. J..

[34] Wenger MPE, Bozec L, Horton MA, Mesquida P (2007). Mechanical Properties of Collagen Fibrils. Biophys. J..

[35] Hua F (2004). Polymer Imprint Lithography with Molecular-Scale Resolution. Nano Lett..

[36] Elhadj S, Rioux RM, Dickey MD, DeYoreo JJ, Whitesides GM (2010). Subnanometer Replica Molding of Molecular Steps on Ionic Crystals. Nano Lett..

[37] *Wettability:* John C. Berg (*ed*.). (Marcel Dekker, 1993).

[38] Lee JN, Park C, Whitesides GM (2003). Solvent Compatibility of Poly(dimethylsiloxane)-Based Microfluidic Devices. Anal. Chem..

[39] Chaudhury MK, Whitesides GM (1991). Direct measurement of interfacial interactions between semispherical lenses and flat sheets of poly(dimethylsiloxane) and their chemical derivatives. Langmuir.

[40] Gates BD, Whitesides GM (2003). Replication of Vertical Features Smaller than 2 nm by Soft Lithography. J. Am. Chem. Soc..

[41] Azatov, M., Sun, X., Suberi, A., Fourkas, J. T. & Upadhyaya, A. Topography on a subcellular scale modulates cellular adhesions and actin stress fiber dynamics in tumor associated fibroblasts. *Phys*. *Biol*. (2017) 10.1088/1478-3975/aa7acc 28635615

[42] Ketchum CM (2015). Actin Dynamics and Signaling Activation of B Lymphocytes Respond to Substrate Topography. Biophys. J..

[43] McCann CP, Rericha EC, Wang C, Losert W, Parent CA (2014). Dictyostelium Cells Migrate Similarly on Surfaces of Varying Chemical Composition. PLoS ONE.

