# Supplementary material for: Replication of biocompatible, nanotopographic surfaces
Source: Sci Rep. 2018 Jan 12;8:564. doi: 10.1038/s41598-017-19008-z (PMC5766624; doi:10.1038/s41598-017-19008-z)
Supplement: Supplementary file 1 — Supplementary information [file 41598_2017_19008_MOESM1_ESM.pdf]

Supplementary Figures for:

Replication of biocompatible, nanotopographic surfaces

Xiaoyu Sun[1], Matt J. Hourwitz[1], Eleni M. Baker[1], B. U. Sebastian Schmidt[2], Wolfgang Losert[2,3,5*], John T. Fourkas[1,2,4,5,*]

[1]Department of Chemistry and Biochemistry, University of Maryland, College Park, MD 20742
[2]Institute for Physical Science and Technology, University of Maryland, College Park, MD 20742
[3]Department of Physics, University of Maryland, College Park, MD 20742
[4]Center for Nanophysics and Advanced Materials, University of Maryland, College Park, MD 20742
[5]Maryland NanoCenter, University of Maryland, College Park, MD 20742

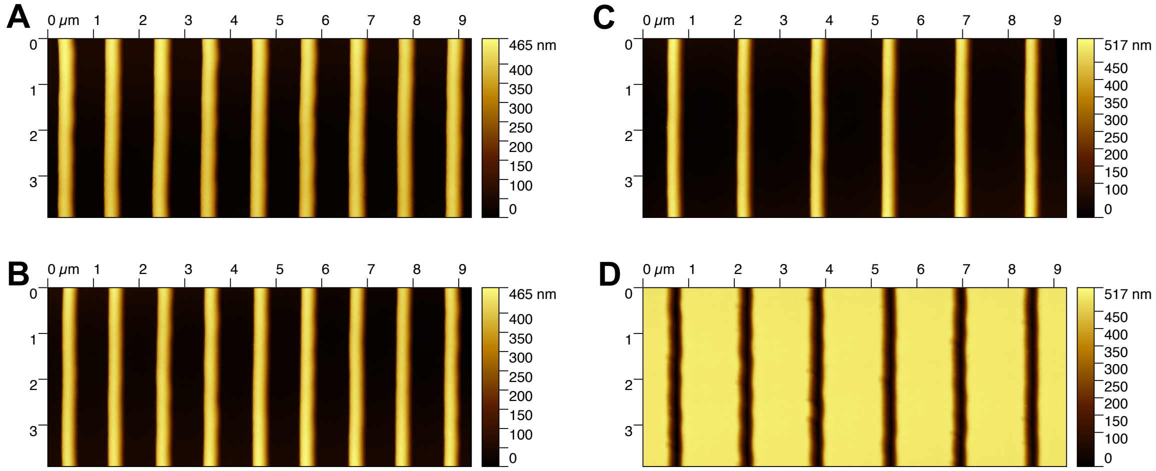

Supplementary Figure 1. AFMs of 1-µm-spaced master (**A**) and replica (**B**) ridges, and 1.5-µm-spaced replica ridges (**C**) and grooves (**D**).

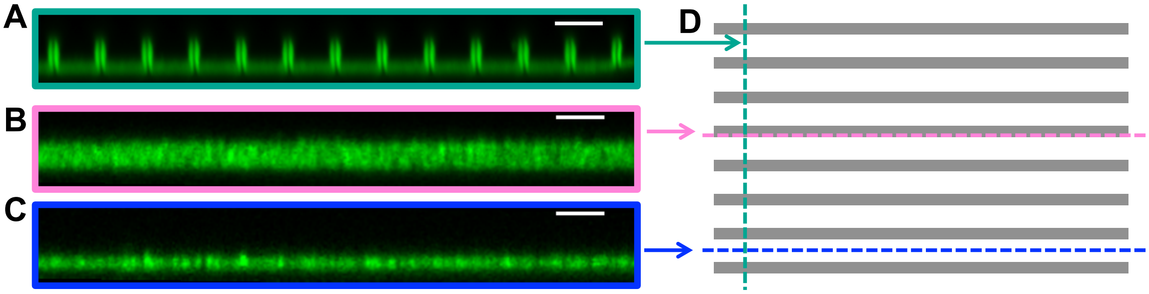

Supplementary Figure 2. Functionalization of nanoridges with fluorescently labeled fibronectin. (**A-C**) Side-view images of the fluorescent-fibronectin-coated nanoridges reconstructed from z-stack confocal images. The planes are perpendicular to the surface in the position of the dashed lines in (**D**) of the color of the image borders. Scale bars, 3 µm.

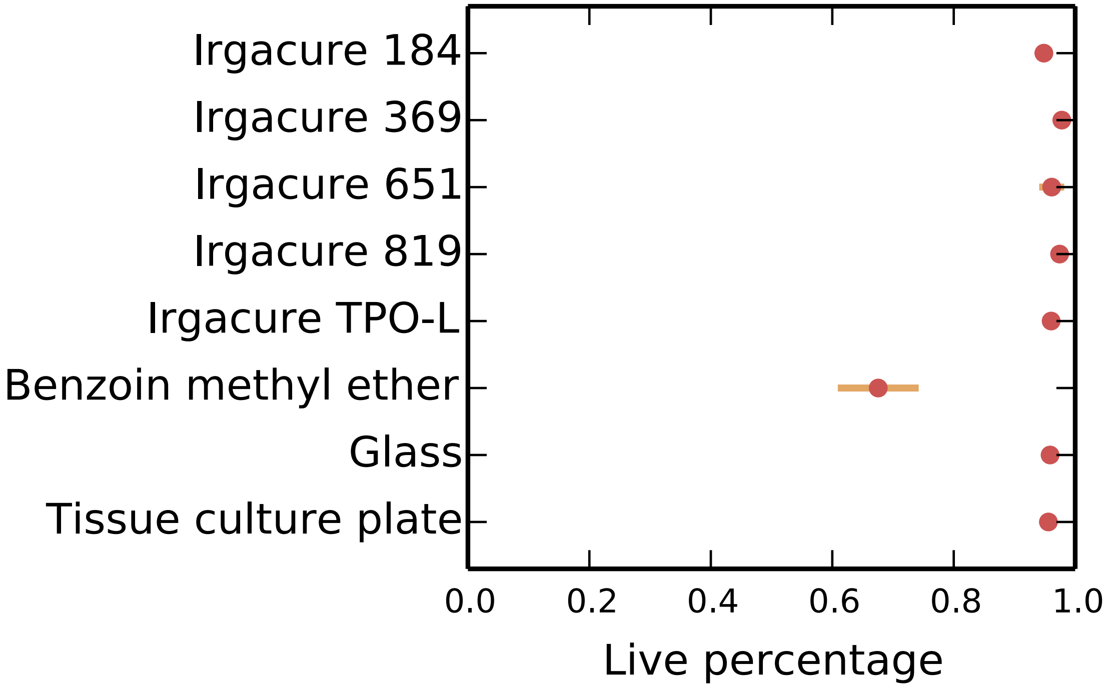

Supplementary Figure 3. Live percentages of cells plated on nanoridges replicated with various photoinitiators. Error bars represent the standard error of the mean.
